# Effect of dental headlights spectrum on the polymerization and working time of light-cured resin composites

**DOI:** 10.4317/jced.59628

**Published:** 2022-06-01

**Authors:** Mateus-Garcia Rocha, Marc-Edward Ottenga, Panagiotis Zoidis, Stefany Pontes, Andre-Figueiredo Reis, Dayane Oliveira

**Affiliations:** 1 Center for Dental Biomaterials, Department of Restorative Dental Sciences, College of Dentistry, University of Florida, Gainesville, FL, USA

## Abstract

**Background:**

The use of dental headlights is a common practice to better illuminate the operatory field and achieve excellence in restorative dentistry. However, visible light-cured dental materials can have reduced working time under headlight illumination. The aim of this study was to evaluate the influence of the spectral irradiance power of two dental headlights on the degree of polymerization and working time of light-curable dental composites.

**Material and Methods:**

Two headlights, StarLight Nano 3 (StarMed) (SN) and Zeon Endevour XL (Orascoptic) (ZE) were characterized using a spectrophotometer coupled to an integrating sphere (MARC® Light Collector, BlueLight Analytics). The degree of conversion of the two composites, Filtek Supreme (3M) and Tetric Prime (Ivoclar Vivadent), was evaluated using an FTIR spectroscope (NicoletTM iS20, Thermo Fisher).

**Results:**

Both headlights emitted a significant amount of blue light. The Zeon headlamp without filter emitted a broader spectrum with lower blue intensity and higher CRI than the White LED of the Nano 3. The Zeon headlamp with the blue blocking filter emitted a broader spectrum than the Orange LED of the Nano 3. There were no differences in the degree of conversion and working time of the Filtek Supreme and Tetric Prime composites when illuminated by the different headlamps. Both Zeon and the White LED of the Nano 3 were capable to cure the composites within only 5-10 minutes of irradiation. There were no changes in the degree of conversion of the composites when the Orange LED of the Nano 3 or the blue blocking filter of the Zeon were used.

**Conclusions:**

Both headlights reduced the working time of light-cured materials. The use of orange filters prevented the composite polymerization and maintained the working time.

** Key words:**Surgical Headlight, degree of conversion, working time, light-curing.

## Introduction

Misdiagnosis and mistreatment are often caused by the inability of the dental practitioner to see the oral cavity in crisp detail. A lot of factors inside the dental treatment room can affect the quality of light (1-3). Fortunately, there are now a few solutions to the everyday concerns of dental practitioners regarding achieving the proper illumination (4,5). One of these solutions is a porTable dental headlight, also referred to as a medical headlight or surgical headlight (5-7).

The dental headlight is porTable and can be conveniently used together with dental loupes when performing various dental procedures (4,5). Dental headlights combined with magnification, provide dentists with a clearer visualization of the oral cavity. This makes it much more possible to precisely identify any dental pathology and perform the most appropriate recommended treatment (1,8,9). However, the improper use of dental headlights during some restorative procedures can affect the properties of light-curable materials, and it can cause deleterious effects on the handling and performance of the restoration (10,11).

Most current resin composites are cured by light in the visible range (12,13). The setting reaction is initiated when the resin composite is irradiated with energy in the visible wavelength spectra. This energy is absorbed by photoinitiators within light-curable material to initiate the polymerization. Light sources other than light-curing units can prematurely initiate the polymerization of light-curable composites. Examples of these light sources are operatory room lights, dental operating lights, sunlight, and dental headlights (14,15). If the resin composite begins to harden prior to the dentist completing insertion and manipulation of the material, it can affect its handling characteristics and increase void formation. It is also likely to affect proper adaptation within the cavity preparation (11).

To avoid premature curing of the composites, manufacturers offer blue-blocking filters (a.k.a. orange shields) that can be coupled to the headlights to filter the light spectrum that can initiate champhorquinone (CQ) polymerization (10,11). Other manufacturers used orange LEDs to prevent irradiation with blue light that can cause CQ photoinitiation. However, there is not much information regarding the spectrum of these Orange LEDs and how efficiently the blue-blocking filters prevent the polymerization of resin-based composite materials (RBCs). Thus, the aim of this study was to evaluate the spectral irradiance power of two dental headlamps, as well as their blue-blocking apparatus and their influence on the working time related to the polymerization of two light-cured resin composites. The null hypotheses tested are:

There will be no differences in the spectral irradiance of the dental headlamps, and the blue-blocking apparatus will not be able to filter the blue light;

There will be no difference in the working time and polymerization of the RBCs exposed to dental headlamps.

## Material and Methods

Two headlights were used in this study (Table 1). The mean irradiances (mW/cm2) of both headlights were measured using a spectrophotometer coupled to an integrated sphere (MARC® Light Collector, BlueLight Analytics, Nova Scotia, Canada). An anodized black aluminum aperture of 10 mm in diameter was placed to limit the integrated sphere area of light entry. The MARC® Light Collector was set to collect the spectrum from 360 – 800 nm, with an integration time of 4 ms and a minimum sensor trigger threshold of 10 mW. The data was collected for all LEDs in different power modes with the headlamp in contact with the sensor. Also, the spectral power was collected for each headlight in the higher power mode with the headlamps 30 cm of distance from the sensor.

**Table 1:**
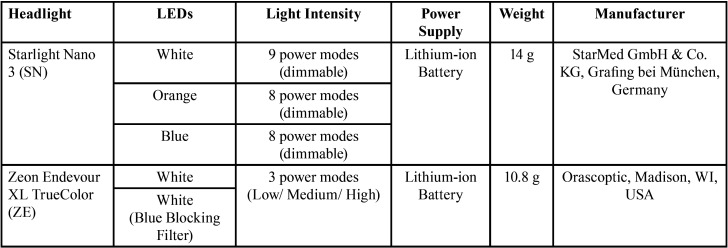
Characteristics of the dental headlights used in this study.

**Figure 1:**
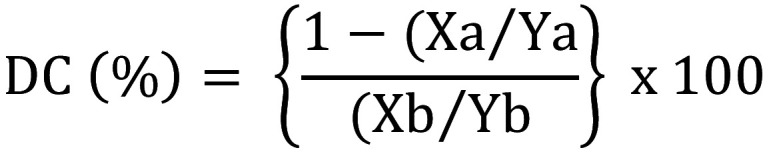
Formula.

Two composites were used in this study: Filtek Supreme (3M, St. Paul, MN, USA) and Tetric Prime (Ivoclar Vivadent, Schaan, Liechtenstein). The degree of conversion (DC) for each composite (n=3) was measured using a Fourier Transformed Infra-Red (FTIR) spectroscope (Nicolet iS20, Thermo Fisher, Waltham, MA, United States). Each composite was placed on a diamond ATR detector of the FTIR spectrometer, a thin polyester sheet was placed on top of the composite, and the composite was compressed to a thin layer (< 0.1 mm) using a glass slide. Then, the material was exposed to one of the different light sources (starLight nano 3 white LED; starLight nano 3 orange LED; Zeon Endeavour XL white LED, and Zeon Endeavour XL white LED with blue-blocking filter) at its maximum intensity at a 30 cm distance from the specimen surface. Infrared spectra between 1700 and 1500 cm-1 were collected before and after 1 min, 5 min, and 10 min of light exposure at 32 scan/min and 4 cm−1 resolution. Unconverted carbon double bonds were quantified by calculating the ratio derived from the aliphatic C=C (vinyl) absorption (1638 cm-1) to the aromatic C=C absorption (1608 cm-1) signals for both polymerized and unpolymerized samples. The DC (%) for the composites was calculated according to the following equation, (Fig. 1):

where Xa (polymerized) and Xb (unpolymerized) represent the bands of the polymerizable aliphatic double bonds, and Ya (polymerized) and Yb (unpolymerized) represent the bands of the aromatic double bonds.

The working time was measured by placing approximately 0.06 g of the material to be tested in the center of a glass microscope slide (75 × 25 mm), placing a second slide on top, and exposing the sample to the light source. The time taken for the material to cease to be homogeneous, assessed as the point at which clefts or voids appeared when the slides were rotated in relation to each other, was measured in seconds using a stopwatch. Each sample was tested three times for different lighting conditions (n=3).

Data were entered into statistical analysis software (Stata/MP 17, StataCorp, College Station, TX, USA) and were checked for normality using Shapiro–Wilk’s test and variance homoscedasticity using Levene’s test. Statistical analyses were performed with a level of significance of α = 0.05. A power analysis was conducted to determine the sample size for each experiment to provide a power of at least 0.8 at a significance level of 0.5 (ß = 0.2). DC was analyzed using a repeated-measures analysis of variance (ANOVA), where the independent variables were set as between-subject groups for the headlights (SN and ZE) and as within-subject groups for the time (1, 5, and 10 min). No direct comparison was made between the composites (Filtek Supreme and Tetric Prime) since different monomer compositions imply different DC.

## Results

**Figure 2:**
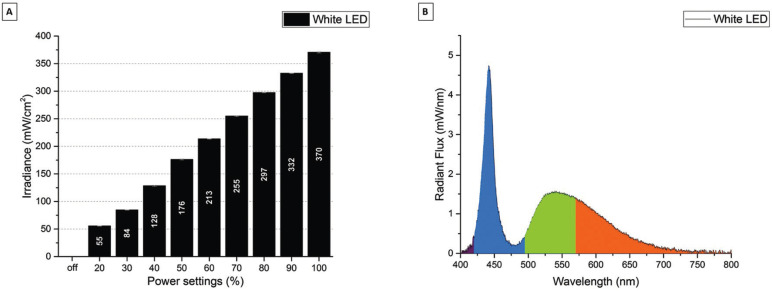
A) Irradiance (mW/cm2) for the Starlight Nano3 White LED in the different power settings. B) Spectral Radiant Flux (mW/nm) for the Starlight Nano3 White LED in the max (100 %) power setting.

Figure 2A shows the irradiance (mW/cm2) for the SN White LED. The SN White LED can be set to 9 different power modes from 55 mW/cm2 to 370 mW/cm2. Figure 2B shows the radiant flux (mW/nm) for the SN White LED. The SN White LED had a peak emission at 442 nm and a broad emission band from 490 nm to 650 nm. The calculated color temperature for the SN White LED was 6561 K.

**Figure 3:**
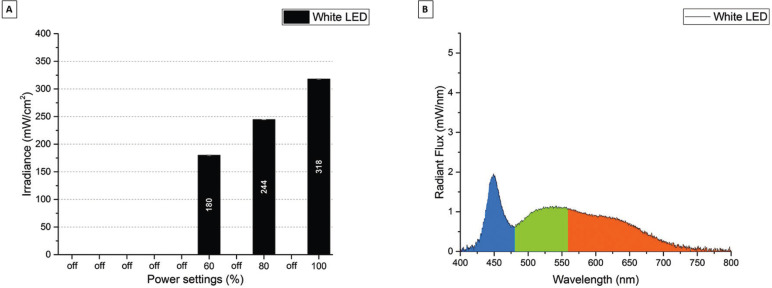
A) Irradiance (mW/cm2) for the Zeon Endevour XL TrueColor White LED in the different power settings. B) Spectral Radiant Flux (mW/nm) for the Zeon Endevour XL TrueColor White LED in the max (100 %) power setting.

Figure 3A shows the irradiance (mW/cm2) for the ZE White LED. The ZE White LED can be set to 3 different power modes from 180 mW/cm2 to 318 mW/cm2. Figure 3B shows the radiant flux (mW/nm) for the ZE White LED. The ZE LED had a peak emission at 448 nm and a broad emission band from 480 nm to 700 nm. The calculated color temperature for the ZE White LED was 6473 K.

**Figure 4:**
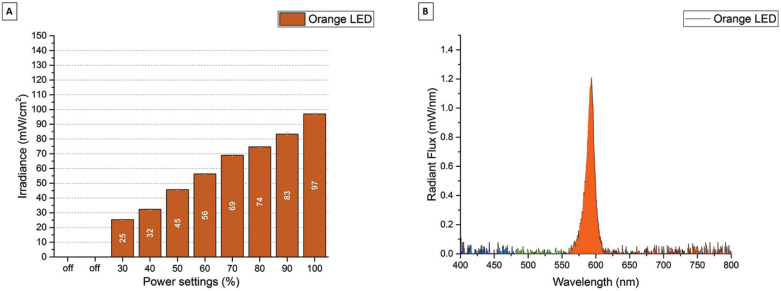
A) Irradiance (mW/cm2) for the Starlight Nano3 Orange LED in the different power settings. B) Spectral Radiant Flux (mW/nm) for the Starlight Nano3 Orange LED in the max (100 %) power setting.

**Figure 5:**
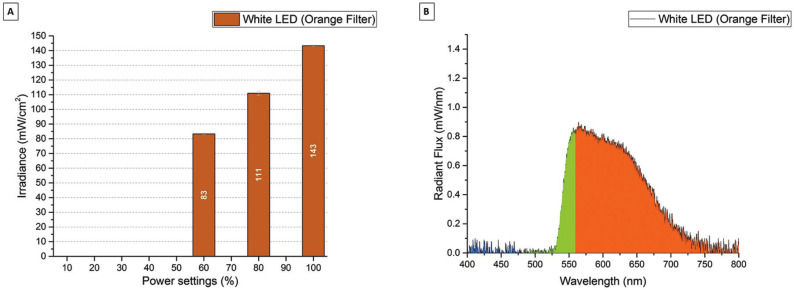
A) Irradiance (mW/cm2) for the Zeon Endevour XL TrueColor White LED with the Orange blue light blocking filter in the different power settings. B) Spectral Radiant Flux (mW/nm) for the Zeon Endevour XL TrueColor White LED with the Orange blue light blocking filter in the max (100 %) power setting.

Figure 4A shows the irradiance (mW/cm2) for the SN Orange LED. The SN Orange LED can be set to 8 different power modes from 25 mW/cm2 to 97 mW/cm2. Figure 4B shows the radiant flux (mW/nm) for the SN Orange LED. The SN White LED had a peak emission at 594 nm. Figure 5A shows the irradiance (mW/cm2) for the ZE White LED with the Orange blue light blocking filter. The ZE White LED with Orange Filter can be set to 3 different power modes from 83 mW/cm2 to 143 mW/cm2. Figure 5B shows the radiant flux (mW/nm) for the ZE White LED with the Orange blue light blocking filter. The ZE White LED with Orange Filter had a wideband emission from 530 nm to 700 nm with peak emission at 564 nm.

Table 2 shows the results for the degree of conversion and the working time of each composite when irradiated with either headlight at a 30 cm distance in its maximum intensity. There were no differences in the degree of conversion and the working time of the Filtek Supreme and Tetric Prime composites when illuminated by the different headlights. Both ZE White and SN White were capable of curing the composites within 5 minutes of irradiation. Both the blue blocking filter and the Orange LED blocked the blue light and extended both composites’ working time.

**Table 2:**
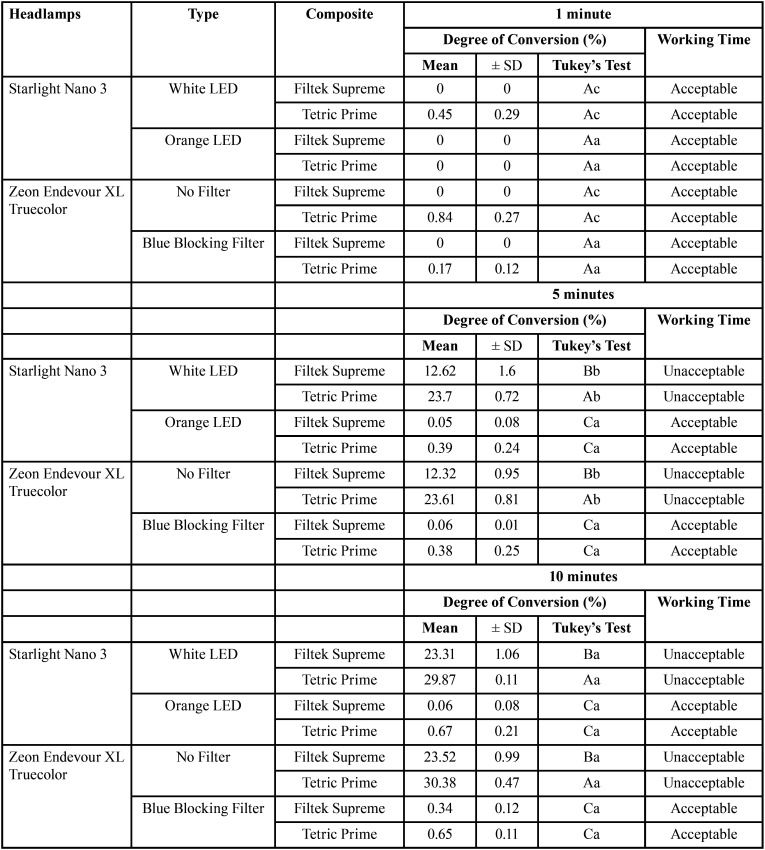
Mean ± SD of the Degree of conversion (DC, %) at different time intervals (1, 5 and 10 min) upon irradiation using the different headlamps with a working distance of 30 cm. * Capital Letter compare means between headlamps (Nano3 and Zeon); Small Case Letters compare mean between irradiation times (1, 5, and 10 min).

## Discussion

The first hypothesis that there would be no differences in spectral irradiance of the dental headlamps and that the blue-blocking apparatus would not be able to filter the blue light was rejected. The dental headlights tested, had slightly different spectral emissions, with SN emitting more blue light than the ZE. These differences are most likely related to the type of white LED used on both dental headlamps. LEDs are made from semiconductor compounds such as Gallium Arsenide (GaAs), Gallium Phosphide (GaP), Gallium Arsenide Phosphide (GaAsP), and Silicon Carbide (SiC), or Gallium Indium Nitride (GaInN), all mixed at different ratios to produce a distinct wavelength of color (16). Different LED compounds emit light in specific regions of the visible light spectrum and therefore have different spectral emissions. The exact choice of the semiconductor material used will determine the overall wavelength of the photon light emissions and, therefore, the resulting color of the light emitted.

There are two approaches to creating white light: Mixed-color and Phosphor-converted. Mixed-color white light approach is to mix the light from several colored LEDs to create a spectral power distribution that appears white. Via a combination of Red, Green and Blue LED chips, the balance of the amount of radiant flux emitted by each of the RGB LED chips is essential to determine the spectral power of the LED and the color temperature. By properly mixing the amount of their output, the resulting light is white in appearance. A phosphor-converted approach to generating white light is using phosphors together with a short-wavelength LED. For example, when one phosphor material used in LEDs is illuminated by blue light, it emits yellow light having a broad spectral power distribution. By incorporating the phosphor in the body of a blue LED with a peak wavelength of around 450 to 470 nanometers, some of the blue light will be converted to yellow light by the phosphor. The remaining blue light, when mixed with the yellow light, results in white light. New phosphors are being developed to improve color rendering. Based on the results of this study, both ZE and SN use Phosphor-converted white LEDs. Nevertheless, the ZE has lower blue emission than SN, likely due to the balance of the semiconductor used.

However, no differences were found in the spectral irradiance when comparing the blue-blocking apparatus. Both the blue-blocking filters and the orange LED efficiently reduced the amount of blue light emission. Previous studies have shown that blue-blocking filters are very efficient in blocking the transmission of blue light by at least 97% (17). In this case, the orange LED is likely to be a GaAsP semiconductor with a Vf at 20mA of 2.0 V. Since these semiconductors only emit light in the wavelength range of 605 – 620 nm, there is no risk of an exciting initiator for the chemical reaction.

The second hypothesis, that there would be no difference in the working time and polymerization of the RBCs exposed to the different dental headlamps, was accepted. ISO 4049 specifies a value that a light-cured material must meet to be considered adequately resistant to ambient light (14,18). The ISO 4049 test for ambient light sensitivity currently uses a xenon lamp, or alternate source with equivalent performance, with appropriate filters to simulate the light spectrum from a dental operating light. The results of one study showed that the working time as determined when mimicking normal dental, the current ISO standard of 8,000 lux, significantly overestimated the “real world” working time of the resin composites. According to the CIE 2008, 1 mW/cm2 is equal to 1,160 lux. Thus, ZE on its high-power emits 370 mW/cm2, equivalent to 429,200 lux. This is approximately 18 times more than the ISO test. For SN the emission is 318 mW/cm2, equivalent 368,880. This is approximately 15 times more than the ISO standard. However, the ISO 4049 tests are designed to test ambient light, and the results show that the headlamp delivers more energy than the regular dental operatory. Thus, the use of white light from headlamps on the composite is critical for the polymerization and working time.

In this study, two composites containing different photoinitiator systems were used: Filtek Supreme contains a Norrish type II photoinitiator system, CQ associated with EDMAB (tertiary amine) (12,13); Tetric Prime has a combination of three photoinitiator systems: a Norrish type II photoinitiator system, CQ associated with EDMAB (tertiary amine); and two Norrish type I photoinitiator systems, TPO and benzoyl germanium (Ivocerin). CQ and the EDMAB photoinitiator system absorb light in the blue range from 420 to 495 nm, with a peak absorption at 470 nm. Ivocerin absorbs light in the violet and blue ranges from 370 to 510 nm, with a peak absorption at 418 nm. TPO absorbs light in the violet range from 350 to 420 nm, with a peak absorption at 370 nm (19-21). However, the results show that the different initiators did not behave differently upon light irradiation from the dental headlight, and the sensitivity to the dental headlight between the two composites can be considered equal.

Trying to work and focus on an area with insufficient light will force the eyes to exert more effort and try harder to have a better vision (22,23). This can cause eye strain or eye fatigue. Dental headlights make it possible for the eyes to work more relaxed (10). Notably, the use of blue-blocking apparatus is essential to ensure the working time of the composites. If the resin composite begins to harden prior to the dentist completing insertion and manipulation of the material, it can affect its handling characteristics, increase void formation, and maladaptation into the cavity preparation (14,15). Thus, when working with composites, the use of orange light is highly recommended to keep the eyes in a more comfortable condition and avoid potential alterations to the properties of visible light-cured materials.

This study showed how important it is to know different dental headlight spectral characteristics and their influence on light-cured composites’ polymerization and working time. It is worthwhile to mention that further studies using a wider variety of dental headlamps are needed.

## Conclusions

Within the limitations of this study, it was possible to conclude that both headlights tested, StarLight Nano 3 and Zeon Endevour XL, reduced the working time of light-cured materials. However, the use of orange light/filters prevented the polymerization of the composites and their working time.
